# Song of the First Lord

**Published:** 1909-02

**Authors:** 


### EDITORIAL DEPARTMENT.

**SONG OF THE FIRST LORD.**—*Pinafore.*

(With Apologies to Gilbert.)

Air: *"Captain of The King's Na-vee."*

Chorus *by the Trustees and Councilors* (*Aside to each other*).

When I was a young man I came across the sea,—
I procured a certificate in home-opa-thee.
I practiced that pathy all simple and pure,
But later caught on to a wa-ter-cure.
I worked that racket in such a clever way
That now I'm the Captain of The A. M. A.

Chorus (*Aside*).

He worked that racket in such clever way
That now he's the Captain of The A. M. A.

The only journal that ever I saw
Was the Lincoln *State Journal of Ne-bras-kaw.*
I plied it with my little ad. in such a catchy style
That I raked in the shekels in a great big pile.
I got so proud that I strutted like a colonel,
And *now* I'm the editor of the A. M. *Journal.*

Chorus (*Aside*).

He got so proud that he strutted like a colonel,
And *now* he's the editor of the A. M. *Journal.*

I moved to the Windy City early in the fall;
I opened up an office and sent around a call.
Those "ethical" fellows soon rallied 'round me,
And the friends that I made was amazin' for to see.
I raised such Cain,—and made such a fuss,
That now I'm Captain of The Oc-to-pus.

Chorus (*Aside*).

He raised such Cain and made such a fuss,
That *now* he's Captain of The Oc-to-pus.

(*Applause in the gallery and music on the tom-tom.*)

Now, homeos, all, wherever you may be,—
If you wish to climb up to the summit of the tree;
If your soul isn't fettered to an ethical creed,
Throw ethics to the devil and follow my lead;
*Organize a Trust* and some fine day
You'll find yourself Secretary of the *A. M. A.*

(*Cat-calls.*) *Exeunt omnes.*
*Curtain.*

